# Proteome characterization of used nesting material and potential protein sources from group housed male mice, *Mus musculus*

**DOI:** 10.1038/s41598-019-53903-x

**Published:** 2019-11-26

**Authors:** Amanda J. Barabas, Uma K. Aryal, Brianna N. Gaskill

**Affiliations:** 1 Department of Animal Science, Purdue University, West Lafayette, IN 47907 USA; 2 Purdue Proteomics Facility, Purdue University, West Lafayette, IN 47907 USA

**Keywords:** Animal behaviour, Proteomics

## Abstract

Laboratory mice (*Mus musculus)* communicate a variety of social messages through olfactory cues and it is often speculated that these cues are preserved in nesting material. Based on these speculations, a growing number of husbandry recommendations support preserving used nests at cage cleaning to maintain familiar odors in the new cage. However, the content of used nesting material has never been chemically analyzed. Here we present the first comprehensive proteome profile of used nesting material. Nests from cages of group housed male mice contain a variety of proteins that primarily originate from saliva, plantar sweat, and urine sources. Most notably, a large proportion of proteins found in used nesting material belong to major urinary protein (“MUP”) and odorant binding protein (“OBP”) families. Both protein families send messages about individual identity and bind volatile compounds that further contribute to identity cues. Overall, this data supports current recommendations to preserve used nesting material at cage cleaning to maintain odor familiarity.

## Introduction

Mice (*Mus musculus*) are the most common species used in research and rely heavily on olfactory signals for communication^1^. Pheromones, a well-known type of olfactory signal, are defined by their ability to reliably trigger specific behavioral and/or physiological responses in their recipients^2^. In fact, most of our current knowledge of pheromone signals is biased toward rodent species: 35 of the 62 known mammalian pheromones originate in rats or mice^3^. Mice can release a variety of compounds in response to various stimuli or social situations which ultimately trigger physical or behavioral responses in their cagemates^2,4–6^. Most odor signals are classified as volatile organic compounds (VOCs)^3^, but protein/peptide signals also play an important role in chemical communication. Several exocrine gland secreting peptides (ESP) from the lacrimal gland influence sexual behavior by triggering lordosis in females and deter unwanted advances towards juvenile males^3^; major histocompatibility complex peptides are crucial for conspecific recognition and mate selection; and several members of the major urinary protein (MUP) family contribute to individual recognition and male dominance status^2,7^. Specifically, MUP20 (also known as “darcin”) not only binds known VOC pheromones, but plays a crucial role itself in learning an individual’s unique VOC profile for mating or general recognition^8,9^. MUP20 has also been shown to promote aggression and indicate social dominance in wild derived and outbred male mice^10,11^. It has been argued that genetic homogeneity may reduce MUP20′s impact on inbred strains, but results similar to wild mice have been reported in C57BL/6 males^12,13^. These effects support the argument that the behavior of any mouse strain can be influenced by odors within a single cage.

Natural mouse behavior drives them to build nests even in the laboratory setting^4^ and it has been suggested that the nest acts as a depository for cage level olfactory signals^14^. In fact, it is commonly suggested for vivarium technicians to preserve the old nest site during cage cleaning in order to maintain existing odor cues in the new cage^15^. However, to date, no one has examined the chemical profile of the nest to confirm if odorants are deposited there and how they may affect research parameters.

Typical nesting behaviors involve manipulating the material using the mouth or paws^16^, so it is expected that the material could hold contents from salivary and plantar glands. Saliva is a known source of several androgen and odorant binding proteins used for individual recognition^17–19^ while the plantar glands produce an oily, sweat-like, substance that has been attributed to a variety of signaling roles such as stranger/conspecific recognition, and route tracing in new territories^14,20,21^. These messages do not change over time and have a lower metabolic cost to the sender if they are long lasting. Therefore, the contents are likely nonvolatile in order to remain stable in the environment^6^. Like nesting material, the contents of plantar sweat have not been analyzed. Additionally, urinary proteins may also be present in the nesting material. It has been reported that mice prefer to keep their nests free of urine and feces^22,23^, but it is possible for them to track urinary compounds onto the material due to limited cage space. The above fluids are all plausible sources of nest chemosignals either from direct material manipulation or random tracks. However, to best understand the messages that may be relayed by these signals, we need to know where they originate and how they are deposited.

A group of 5 mice, a typical laboratory housing density, in a standard sized shoebox cage has the potential to create a complex odor environment that may influence physiological and behavioral measures. However, two odor sources, nesting material and sweat, have not been the subject of chemical profiling. Therefore, the objective of this study was to characterize the protein profile of used mouse nesting material. We then compared the nest’s proteome to that of plantar sweat, saliva, and urine for a more comprehensive overview of its content’s plausible origins.

## Results

To assess the proteome content of nesting material, we housed 8 week old male C57BL/6NCrl mice in groups of five with 8.5 g of crinkle paper nesting material. This form of material allows the mice to build more complex, naturalistic nests^24^. We chose to collect nest samples after one week because that is a common length in between cage cleaning for static housing across animal facilities. Commonly, facilities completely replace the nest with new material at cage cleaning, so our samples represent a maximum amount of protein content to which the mice would be exposed. To trace the nest profiles to tentative protein sources, we collected sweat and saliva samples the same day as nest collection while urine samples were collected two days prior. Proteins were extracted from all four sample types, underwent trypsin/LysC digestion, and were analyzed using liquid chromatography- tandem mass spectroscopy (LC- MS/MS).

### Global quantitation

**Figure 1:**
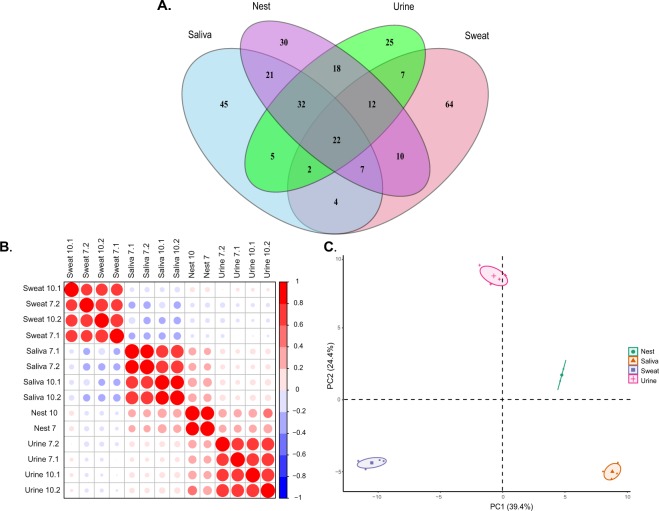
Profile analysis of nesting material, sweat, saliva, and urine proteomes. (**A**) Venn diagram of proteins quantified with at least 2 peptide counts in 2 replicates of a single sample type. (**B**) Pearson Correlation plot between replicates based on hierarchical clustering of log2 label free quantitation (LFQ) intensities. (**C**) Principal component analysis sample plot based on log2 LFQ intensities of 140 common proteins detected in at least two sample types; percentages in parentheses represent the explained variance for the first and second Principal Component (PC). See Supplementary Table 2 for complete list of protein loadings.

We detected 432 proteins/protein groups across all sample types and filtered that list to the 304 proteins with at least two MS/MS peptide counts per protein in at least 2 replicates of a single sample type. Of that list, 46% (140) were common to at least 2 sample types; 10% were unique to the nest samples; 21% were unique to sweat samples; 15% were unique to saliva samples; and 8% were unique to urine samples (Fig. 1A). Comprehensive peptide and protein lists and quantifications can be found in the Supplementary Information (Table S1). Pearson correlation coefficients of log_2_ label free quantitation (LFQ) were highest within sample type (Fig. 1B). Nest sample replicates had a correlation coefficient of 0.85. Average coefficients between sweat, saliva, and urine replicates were 0.69, 0.80, and 0.76 respectively. Nest samples also showed coefficients of at least 0.2 with saliva and urine samples, but had minimal correlation with sweat samples. There was also a slight negative correlation between sweat and saliva samples, with coefficients less than −0.2 between most replicates. A principal component analysis (PCA) was used on log_2_ LFQ intensities for the 140 common proteins present in at least two sample types. It showed that replicates for each sample type cluster together on PC1 and PC2 (Fig. 1C). Variation on PC1 separated all sample types while variation on PC2 separated urine and nest samples from saliva and sweat. Individual protein loading values for PC1 and PC2 are listed in Table S2.

### Chemosensory related expression patterns

**Figure 2:**
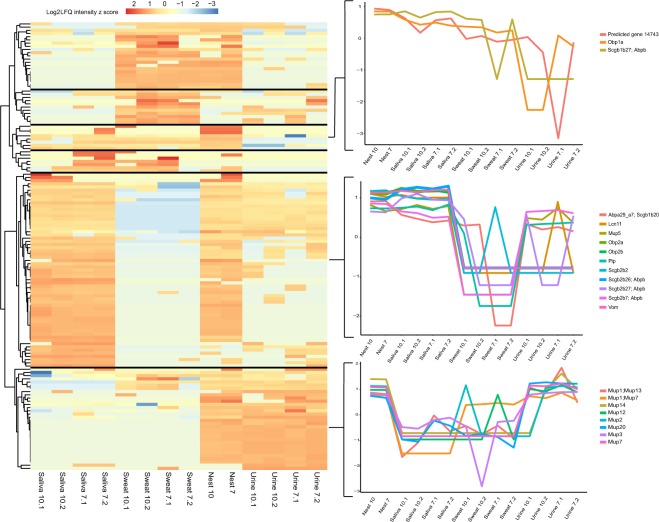
Protein abundance varies across sample types. The heatmap depicts the change in intensity for 140 proteins found in at least two different sample types. Hierarchical clustering was used to classify the proteins into six clusters. The color scale depicts log2(LFQ intensity) z-scores. Expression patterns for 21 peptides with known odor functions are emphasized in the line graphs.

The 140 common proteins were grouped into six clusters using hierarchical clustering based on log_2_ LFQ z-scores (Fig. 2). Twenty seven of these common proteins were matched to known genes with chemosignal or odorant binding function (Table 1) and were primarily found in three of the six protein clusters (Fig. 2). Six proteins matched to members of secretoglobin (Scgb) family and were primarily androgen-binding protein (ABP) subunits. They showed high abundance in saliva and nest samples and overall, had low abundance in sweat samples with the following exceptions: Scgb1b27 had high abundance in two sweat replicates while Scgb2b2 had high abundance in one sweat replicate. Scgb proteins also had low abundance in urine samples with the exception of Scgb2b27 which had high abundance in two replicates and Scgb2b7 which had high abundance in all urine replicates (Fig. 2 inset).

**Table 1:** Proteins with odorant related functions based on Gene Ontology (GO) searches.

Common Proteins		
Protein ID	Protein name	Gene name
Q8R1E9; Q7TNY5	ABPBG27; Salivary androgen-binding protein beta subunit	Scgb2b27
Q3UU48; P02816	Prolactin-inducible protein homolog	Pip
A2ANT5; P11590	Major urinary protein 4	Mup4
Q9D3H2	Odorant-binding protein 1a	Obp1a
A2BHD2	Predicted gene 14743	Gm14743
O35176	Androgen binding protein A2	Scgb1b2
Q58ES8; A2CEL1	Major urinary protein 1	Mup1; Mup13
D2XZ31; E9PWZ2	Androgen binding protein A7; A20	Abpa29_a7; Scgb1b20
A2BIN1; Q4FZE8	Major urinary protein 10; Major urinary protein 1	Mup10; Mup1
Q5FW60	Major urinary protein 20	Mup20
Q3KQQ2; P04939	Major urinary protein 3	Mup3
Q91WB5; G3UXN8	Androgen binding protein A27	Scgb1b27
D2XZ37; G5E8B4	Secretoglobin family 2B member 2	Scgb2b2
P11591	Major urinary protein 5	Mup5
D3YYY1	Androgen binding protein BG7	Scgb2b7
A2BHR2	Lipocalin 11	Lcn11
P11589	Major urinary protein 2	Mup2
Q58EV3; E9QA79	Major urinary protein 1; Major urinary protein 7	Mup1; Mup7
A2CEK7	Major urinary protein 12	Mup14
Q8JZX1; Q7M745	Androgen binding protein BG26	Scgb2b26
Q8K1H9	Odorant-binding protein 2a	Obp2a
A2BHR0	Odorant-binding protein 2b	Obp2b
Q80XI7	Vomeromodulin	Vom
D2XZ39; Q7M747	Secretoglobin family 2B member 24	Scgb2b24
A8R0U8; A8R0U7	Exocrine gland secreted peptide 15	Esp15
L7MUC7	Major urinary protein 7 (Fragment)	Mup7
B8JI96	Major urinary protein 14 (Fragment)	Mup14
**Unique Proteins**		
Protein ID	Protein name	Gene name
**Saliva**		
Q24JQ8; Q62472	Vomeronasal secretory protein 2	Lcn4
Q14AJ3; Q62471	Vomeronasal secretory protein 1	Lcn3
G5E8B5; Q7M742	Secretoglobin family 1C member 1	Scgb1c1
**Nest**		
J3QK77; Q9JI02	Secretoglobin family 2B member 20	Scgb2b20
A8R0U0	Exocrine gland secreted peptide 6	Esp6
J3QJY4	Androgen binding protein A3	Scgb1b3
S4R2L0; J3QM75	Androgen binding protein BG12; Androgen binding protein BG19	Scgb2b12; Scgb2b19
Q9D3N5	RIKEN cDNA 5430402E10 gene	5430402E10Rik
S4R1X8; S4R2V3	Secretoglobin, family 2B, member 17; member 15	Scgb2b17; Scgb2b15
A0A089N3F1; D2XZ38	Androgen binding protein BG3	Abpbg3; Scgb2b3
**Urine**		
A9R9V7	Major Urinary Protein 21	Mup21
A2CEK6; L7N222	Major urinary protein 11; Major urinary protein 13	Mup13

Detected proteins had at least 2 MS/MS counts in two replicates of a single sample type. List is limited to the first two protein IDs where applicable and organized by proteins common to at least two sample types and those unique to each sample type.

Peptides from several lipocalins were also detected across sample types and may function as pheromone transporters. Three odorant binding proteins (OBP) had high abundance levels in saliva and nest samples and variable sweat and urine presence. Obp2a and Obp2b peptides had low abundance levels in sweat and urine samples while peptides from Obp1a had high abundance in sweat and variable abundance in urine samples (Fig. 2 inset). Additionally, vomeromodulin and lipocalin11 had high abundance in nest and saliva samples and low abundance in sweat and urine samples. However, lipocalin11 had high abundance in one urine replicate (Fig. 2 inset).

Nine MUP proteins, including MUP20, were also detected across all sample types. These peptides had high abundance in nest and urine samples and low abundance in saliva except for MUP5 which had high abundance in saliva samples. Overall, MUP expression in sweat samples was low with the following exceptions: peptides for MUP1; MUP7 had high abundance in sweat; peptides for MUP12 and MUP2 had high abundance in one sweat replicate respectively (Fig. 2 inset).

Peptides for MUP4 and Scgb2b24 were also detected in nest samples and had low abundance in sweat samples. Both had variable abundance in saliva. In urine, MUP4 had variable expression while Scgb2b24 was low (Fig. S1).

ESP15 peptides had high abundance in nest samples, but only had high abundance in one saliva replicate (Fig. S1).

MUP10; MUP1 peptides had high abundance in all samples, but had low abundance in one saliva replicate (Fig. S1).

Chemosignal peptides unique to each sample type are also listed in Table 1. In summary, submaxillary gland protein 3A and vomeronasal protein 2 were detected in all saliva replicates while vomeronasal protein 1 and Scgb1c1 were detected in two saliva replicates. MUP21 was present in all urine replicates while MUP11 was present in one urine sample. Sweat samples did not contain any unique known odor related proteins. Both nest samples contained four Scgb proteins, submaxillary gland protein 2, ESP6, and cDNA gene 5430402E10 with predicted odor carrier properties.

### Protein functions

**Figure 3:**
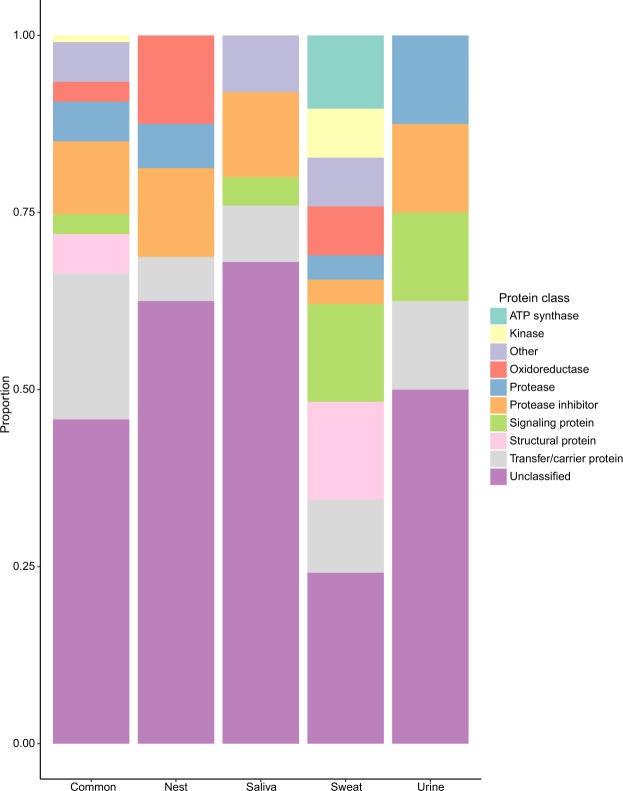
Functional classification of common and unique proteins. GO category proportions of proteins found in at least 2 sample types (common) and unique to each sample type. Proteins were only included if their protein IDs were matched in the PANTHER database. Proteins were considered “Unclassified” if the GO search did not provide a listed category.

Of the 273 detected proteins, 68% were annotated in the Gene Ontology (GO) database based on cellular molecular function. Transfer/carrier proteins, which can bind odorants, account for approximately 21% of common proteins; 6% of unique nest proteins; 10% of unique sweat protein; 13% of unique urine proteins protein; and 8% of unique saliva proteins (Fig. 3). Signaling proteins, which may act as chemosignals themselves, account for approximately 3% of common proteins; 14% of unique sweat proteins; 13% of unique urine proteins; and 4% of unique saliva proteins (Fig. 3).

### Most abundant proteins

**Figure 4:**
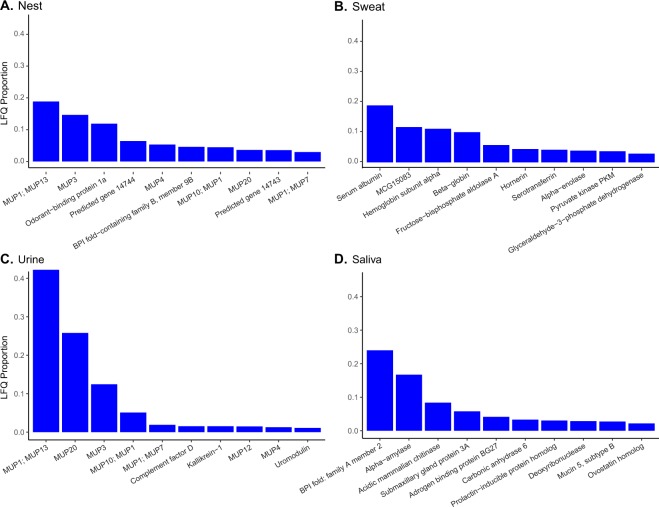
Top ten most abundant proteins in each sample type expressed as a proportion of total LFQ intensity across individual samples for (**A**) nesting material, (**B**) sweat, (**C**) urine, and (**D**) saliva.

Based on the proportion of LFQ intensities, six of the top ten proteins in nest samples are members of the MUP family, accounting for just under 50% of total protein abundance in the nest site. Approximately 15% of nest site peptides were matched to Obp1a or predicted gene 14743, which has an estimated carrier protein role (Fig. 4A). None of the top ten proteins in sweat samples have a known odorant association role (Fig. 4B). Seven of the top ten urinary proteins are members of the MUP family accounting for over 90% of total proteins in urine samples (Fig. 4C). Three of the top ten saliva proteins had odorant related functions (ABP BG27, submaxillary gland protein 3A, and prolactin inducible protein) and account for 13% of total saliva proteins (Fig. 4D).

## Discussion

Although there is a growing effort to consider how the environment may impact laboratory animal well-being and data reproducibility, the olfactory environment is not given appropriate consideration. In mice, preserving used nesting material has been shown to reduce aggression in males^14^ and is suggested as part of standard husbandry to preserve odor cues^15^. However, this is the first report to identify and quantify deposits on the nesting material and other sources to determine the origin of the deposits. Our analyses show that after one week in the mouse cage, nesting material acquires a variety of chemosignal proteins from sweat, saliva, and urine sources. Additionally, nest samples contain unique proteins that may originate from sebaceous glands, other oro-facial glands, or fecal residues. Mice prefer to defecate and urinate away from the nest site^23^, but due to the restricted area in a standard mouse cage, urine and feces likely enter the nest due to regular activity. This data provides evidence of urinary proteins in the nest, although we did not record where the mice chose to urinate in relation to the nest site.

Overall, the nest site contains a variety of proteins used by mice for identification. This supports the rationale behind preserving nesting material to maintain familiar scent marks^15^. To start, the most prevalent proteins in the nest, accounting for approximately half of the total abundance, belong to the MUP family. While MUPs are primarily found in urine, these proteins are also found in sweat and saliva. The diverse MUP ratio between individuals serves as an identification mechanism as mice spend the most time investigating urine marks with a different MUP profile than their own^25^. These profiles provide specific information about the signaler such as health and social status^2^. Even though members of the same inbred strains have little diversity in their MUP profiles^26^, maintaining the high abundance of MUPs through nest transfer is still beneficial for mouse welfare. Instead of being placed into an unmarked, odor-free environment at cage change, nest transfer allows the mice to maintain odor familiarity through the deposits in the preserved material.

Additionally, several ABP and OBP/lipocalin proteins were detected in the nest samples. ABP dimers in the saliva help facilitate mate choice in female mice by providing subspecies identification cues^27^. OBPs are known to transport VOCs and are expressed in several oro-facial glands with the protein product ultimately detected in saliva^28^. Since mice typically engage in facial sniffing when initiating social interactions^4^, it has been suggested that these proteins may play a role in chemical communication: the mixture of self and conspecific odor is spread through self-grooming to promote peaceful interactions^19^. The presence of OBPs in the nest site may further expand this hypothesis. Through the act of repeated oral nest manipulation, mice deposit their own OBPs and pick up OBPs from their cage mates. In addition, group sleep in a common nest area may also spread the OBP mixture onto each cage mate’s fur, further promoting peaceful social behavior.

Two members of the ESP family were also detected in nesting material: ESP6 and ESP15. ESP genes are clustered near MHC loci in the mouse genome^29^ and are produced primarily by the lacrimal gland^30^. 14 members of the ESP family, including ESP6 and ESP15, are capable of stimulating neurons in the vomeronasal organ (VNO)^29^. Although the direct function of ESP6 and ESP15 are unknown, they may serve as chemosignals since proper sensory activity by the VNO is necessary to express appropriate sex-specific behaviors^31^ and many known mouse pheromones function through VNO activation^32^. Ultimately, the identification of multiple proteins and potential chemosignals in the nest site is likely a driving factor behind the reduction in male aggression seen when nesting material is preserved at cage change^14^.

Despite the nest’s ability to reduce aggression at cage change, one of its most abundant proteins, MUP20 (“darcin”), elicits male aggression at levels comparable to that of whole urine exposure^33^. However, MUP20 has been shown to play a crucial role in social learning by female mice. Females pre-exposed to urinary MUP20 form a learned attraction to the source male’s VOC profile^34^. It is possible that a similar mechanism occurs in male cages where deposited MUP20 within the nest site stimulates learning of cage mate profiles. It is also possible that MUP20 in the nest may be deposited from a variety of secretions. MUP20 is commonly thought of as a urine component that binds VOC pheromones which promote aggression^35^. However, our data confirms a previous report of MUP20 being present in saliva^19^ and shows that, among several MUP peptides, it is present in sweat as well. MUP20 originating in saliva and sweat may not elicit the same behavioral response as the urinary form since the VOCs it binds are unique to male urine^36^. While recombinant MUP20 can elicit aggression on its own^33^, perhaps MUP20 in saliva and sweat bind a different ligand that reduces its aggression provoking signal. That answer to that question was beyond the scope of this study’s aim.

Pilocarpine was used in this study because sufficient amounts of sweat and saliva could not be collected naturally for analysis. While necessary, it is worth considering the potential impact of the drug on protein data. Pilocarpine induces fluid release by stimulating M3 muscarinic receptors on the sweat and salivary glands^37,38^. Currently, it is not known how pilocarpine stimulation may influence the secreted gland content, but we acknowledge that these samples may not reflect naturally occurring protein ratios. Additionally, all body fluid samples may have been impacted by each mouse’s social status. Sampled mice were chosen based on their dominance ranking, which may have contributed to natural variation between samples. It may also explain variation between protein ratios in the nest compared to other sample types: the nest contains a pooled sample from all mice in the cage, so secretions from dominant and subordinate mice are inter-mixed. Dominant mice are known to produce more MUPs, particularly MUP20, than subordinates^10–12^, but it is unknown whether social ranking influences other protein levels.

Overall, our saliva and urine proteomes contained proteins that were also reported in previous studies. In saliva, we detected several ABP analogs, MUPs, ESPs, Kallikerin-1, OBP analogs, prolactin inducible protein homolog, and amylase that match past reports from C57BL/6J and BALB/c mice^17,18,39^. In urine, a majority of our detected proteins were members of MUP family, which have been well documented in previous reports^26,34,40–42^. In addition, MUPs have been reported in rat urine, with MUP13 displaying pheromone properties, further supporting their role in olfactory communication across species^43,44^.

This initial protein characterization provides a framework for further studies focused on the cage level olfactory environment. Due to the prevalence of proteins that contain identity information, it is probable that the nest profile will vary based on strain, sex, age, and reproductive status. Nest sites from breeder pairs or trios may contain additional signals that strengthen parent-offspring relations. Maintaining familiar odors from the home cage may also prove beneficial when acclimating mice to a new behavioral testing arena. It is also worth examining how the nest contents could change before and after aversive procedures or if the mice are inoculated for an infectious disease study. Situations where the mice become stressed or sick may cause them to produce an aversive signal indicative of danger that should not be preserved in the cage.

More broadly, a recent initiative throughout biomedical science aims to reduce the level of preclinical research that is not reproducible. In a recent survey of the scientific community, 90% of respondents felt there was either a “slight” or “significant” reproducibility crisis in research data^45^. Over 80% of participants also claimed that “selective reporting” and unavailable methods are common factors contributing to the crisis. As an attempt to increase method transparency, the National Centre for the Replacement, Refinement, and Reduction of Animals in Research developed the ARRIVE guidelines for reporting preclinical study procedures^46^. Item 9 of ARRIVE focuses on animal housing and husbandry in which researchers are instructed to report a wide range of environmental parameters for their study animals. This includes housing environment, lighting conditions, and temperature/humidity ranges throughout the study. However, the ARRIVE guidelines fail to acknowledge the animals’ chemical/olfactory environment and many researchers do not consider how their studies may be affected by odors. Findings from this study bring attention to the diverse olfactory environment found in standard mouse cages.

In summary, we present the first proteome characterization of used nesting material from group housed male mice. It is commonly suggested to preserve used nesting material throughout cage changes to preserve the cage level olfactory environment and this study provides quantitative evidence to support this practice. Used material contains a large assortment of proteins, many of which contain identification information. These identity cues likely play a communication role between cage members. Further research is warranted to explore the role between these complex odor profiles and social behavior.

## Methods

Ethics statement: All methods involving animals were approved by the Purdue University Institutional Animal Care and Use Committee (protocol #1707001598) and follow federal animal guidelines.

### Animals

This study used two cages of five male C57BL/6NCrl mice acquired from Charles River Laboratories (Wilmington, MA). All mice were specific pathogen free. Mice were approximately 8 weeks of age upon arrival and housed in open top, 11.5″ × 7.25″ × 4.25″ mirco-isolator cages (Ancare, Bellmore, NY) with aspen wood chip bedding (NEPCO, Warrensburg, NY), 8.5 g virgin kraft crinkle paper nesting material (Enviro-dri, Cleveland, Ohio), and *ad libitum* food (Envigo, Teklad 2018, Indianapolis, IN) and water treated by reverse osmosis. The mice were housed for one week under a 12:12 light: dark cycle between 20.6–22.2 °C with 28–50% relative humidity. These mice were part of a larger, behavioral study.

### Sample collection and protein extraction

Unless otherwise noted, all samples were collected at the end of the weeklong study when mice were approximately 9 weeks of age. All fluid samples were collected from two mice per cage. Those mice were chosen based on their social ranking as determined by the tube test^47^. Briefly, a one inch diameter tube was secured between two plexiglass arenas. After each individual mouse was acclimated to the arena, pairwise trials were conducted between cage mates in which one mouse was placed at each end of the tube and simultaneously released. After meeting in the middle, the first mouse to back out of the tube was declared the trial loser. All trials were replicated four times and the arena was cleaned with ethanol and air dried between trials. Each mouse’s win percentage was determined from the number of trials he won divided by the number in which he competed. The mouse with the highest win percentage in each cage was considered the dominant, while the mouse with the lowest was the subordinate.

#### Nest

One sample of crinkle paper, containing 25 individual strips, was taken from each cage (N = 2), since groups of mice sleep together in a communal nest. The center and periphery of the nest were sampled using metal forceps that had been previously cleaned with acetone and allowed to air dry. Since mice restructure their nests daily^48^, it is unknown whether secretions would be equally distributed throughout the nest.

#### Sweat

Mice were anesthetized with compressed isoflurane throughout the procedure. Sweat samples were collected from two mice per cage (N = 4) by injecting 50 µL of a 1 mg/mL pilocarpine hydrochloride solution (Sigma-Aldrich, St. Louis, MO) subcutaneously to one hindfoot. After losing consciousness, their feet were cleaned with ethanol, allowed to dry, and injected with the pilocarpine solution. The highest volume of sweat is produced approximately 10–20 minutes post injection^49^, so strips of 100% cotton filter paper (Ahlstrom, Helsinki, Finland) were secured to the foot for 20 minutes after injection using plastic hair clips (Conair, East Windsor, New Jersey). The clips held the filter paper in place without compromising blood flow to the foot. After 20 minutes, individual filter paper strips were stored in 1.5 mL centrifuge tubes in a −80 °C freezer until processing. Mice were euthanized following the collection of all the samples.

#### Saliva

The pilocarpine solution used for sweat collection also stimulates saliva production, so the acrylic anesthesia chamber floor was wiped with ethanol after the mice lost consciousness in preparation for saliva collection. Saliva was collected from the same mice used for sweat sampling (N = 4) via pipette and stored in a −80 °C freezer until processing.

#### Urine

On day 5 of the study, urine was collected by scruffing each mouse over a clean bowl lined with aluminum foil. Gentle abdominal massage was applied when needed. Urine was collected from all mice, but only analyzed from mice sampled for sweat and saliva (N = 4). Samples were stored in a −80 °C freezer until processing.

### Sample preparation

Protein samples were prepared for analysis as reported previously^50^. Proteins were extracted from the nesting material and sweat filter paper using a 20 mM TRIS-HCl, pH 7.5 extraction buffer and precipitated with 5x the sample volume of acetone. Proteins in all samples were denatured using 40 µL of 8 M urea and total quantities were determined using a bicinchoninic acid assay. The samples were reduced with 10 mM dithiothreitol, alkylated with 20 mM iodoacetamide, and digested at 37 °C for 5 hours with a mass-spec grade trypsin and Lys-C mix (Promega, Madison, WI) at a minimum 1:25 enzyme to substrate ratio. Peptides were desalted using Pierce C18 spin columns (Pierce Biotechnology, Rockford, IL), eluted with 80% acetonitrile (ACN) and 0.1% formic acid (FA), and dried at room temperature in a vacuum concentrator for 1 hour. Clean, dry peptides were resuspended in 97% purified water, 3% ACN, and 0.1% FA at a final concentration of 0.2 µg/µL.

### Liquid chromatography/ tandem mass spectroscopy (LC-MS/MS)

Samples were analyzed by reverse-phase LC-ESI-MS/MS system using the Dionex UltiMate 3000 RSLC nano System coupled to the Orbitrap Fusion Lumos Mass Spectrometer (Thermo Fisher Scientific, Waltham, MA). Peptides were loaded onto a trap column (300 μm ID × 5 mm) packed with 5 μm 100 Å PepMap C18 medium, and then separated on a reverse phase column (50-cm long × 75 µm ID) packed with 2 µm 100 Å PepMap C18 silica (Thermo Fisher Scientific, Waltham, MA). The column temperature was maintained at 50 °C. All the MS measurements were performed in the positive ion mode, and using 120 min LC gradient and standard data-dependent mode^50^. MS data were acquired with a Top20 data-dependent MS/MS scan method. Instrument was calibrated at the start of each batch run and then in every 72 hours using calibration mix solution (Thermo Fisher Scientific, Waltham, MA). The performance of the instrument was also evaluated routinely using *E. coli* digest.

### LC-MS/MS data analysis

LC-MS/MS data were analyzed using MaxQuant software (version 1.6.3.3) against the UniProtKB *Mus musculus* genome (85,159 sequences as of Feb. 2019, www.unitprot.org). Unless stated otherwise, default settings were used. We edited the following parameters for our search: 10 ppm precursor mass tolerance; trypsin/Lys-C enzyme specificity; variable modification was oxidation of methionine (M); fixed modification was carbamidomethylation of cysteine (C); false discovery rate (FDR) of 0.02; peptide spectral match (PSM) and protein identification was set to 0.01. Label free quantitation (LFQ) was selected. All quantifications were calculated by MaxQuant. After the search, peptides with MS/MS counts under 2 were removed from the dataset. Log2 LFQ values were used for analyses in R Studio (version 3.4.3) with the following packages: *tidyverse, VennDiagram, pheatmap, RColorBrewer, magrittr, corrplot, FactoMineR, factoextra*, and *cowplot*.

### Bioinformatics analysis

All majority protein IDs were searched in the PANTHER gene database (www.pantherdb.org) and compared to the entire verified *Mus musculus* proteome (Swiss-prot, 22,262 proteins, version 14.0 April 2018). In cases where a protein had multiple IDs, only the first two were used in the search. Classification is based on Gene Ontology (GO) for the molecular function category.

## Supplementary information

Supplementary Information

## Data Availability

All raw LC-MS/MS data are available on the Mass Spectrometry Interactive Virtual Environment (MassIVE) data repository at ftp://massive.ucsd.edu/MSV000084022.
