# Supplementary material for: Proteome characterization of used nesting material and potential protein sources from group housed male mice, Mus musculus
Source: Sci Rep. 2019 Nov 26;9:17524. doi: 10.1038/s41598-019-53903-x (PMC6879570; doi:10.1038/s41598-019-53903-x)
Supplement: Supplementary file 1 — Supplementary Information [file 41598_2019_53903_MOESM1_ESM.pdf]

Proteome characterization of used nesting material and potential protein sources from group housed male mice, *Mus musculus*

Amanda J. Barabas, Uma K. Aryal, & Brianna N. Gaskill

Supplementary Information

Supplementary Table 1

**Proteins in all 4 sample types**		
Protein IDs	Protein names	Gene names
P00687;Q99KE6	Alpha-amylase 1	Amy1
B1ARR4	Carbonic anhydrase 6	Car6
A2AJD1	BPI fold-containing family B, member 9B	Bpifb9b
Q8R1E9;Q7TNY5	ABPBG27; Salivary androgen-binding protein beta subunit	Scgb2b27;Abpb
Q3UU48;P02816	Prolactin-inducible protein homolog	Pip
A2ANT5;P11590	Major urinary protein 4	Mup4
Q9D3H2	Odorant-binding protein 1a	Obp1a
Q546G4;P07724	Serum albumin	Alb
A2BHD2	Predicted gene 14743	Gm14743
O88968	Transcobalamin-2	Tcn2
O35176	Androgen binding protein A2	Scgb1b2
Q58ES8;A2CEL1	Major urinary protein 1	Mup1;Mup1
D2XZ31;E9PWZ2	Androgen binding protein A7; A20	Abpa29_a7;Scgb1b20
Q921I1;E9Q035	Serotransferrin	Tf;Gm20425
A2BIN1;Q4FZE8	Major urinary protein 10; Major urinary protein 1	Mup10;Mup1
Q5FW60	Major urinary protein 20	Mup20
E9QNP3;F8WJ23	Hornerin	Hrnr
A8DUK4;A8DUK7	Beta-globin	Hbbt1;Hbb-bs
Q3UAF7;Q3UAF6	Actin, cytoplasmic 1	Actb;Actg1
D2KHZ9;A0A0A0MQF6	Glyceraldehyde-3-phosphate dehydrogenase	GAPDH
Q58E64;Q3UA81	Elongation factor 1-alpha 1;Elongation factor 1-alpha 2	Eef1a1;Eef1a2
Q3KQQ2;P04939	Major urinary protein 3	Mup3
**Proteins in 3 sample types**		
Protein IDs	Protein names	Gene names
**Saliva, Sweat and Nest Proteins**		
Q91WB5;G3UXN8	Androgen binding protein A27	Scgb1b27
A2AEN9	Predicted gene 5938	Gm5938
D2XZ37;G5E8B4	Secretoglobin family 2B member 2	Scgb2b2
Q5FW97;P17182	Alpha-enolase;Enolase	EG433182;Eno1
Q08189	Protein-glutamine gamma-glutamyltransferase E	Tgm3
Q3UEK9;Q3UEK5	Alpha-2-HS-glycoprotein	Ahsg
Q91X72	Hemopexin	Hpx

**Saliva Sweat and Urine Proteins**		
Q564E2;Q3TI99	L-lactate dehydrogenase; L-lactate dehydrogenase A chain	Ldha
P06745;B2RXT5	Glucose-6-phosphate isomerase	Gpi;Gpi1
**Saliva Nest and Urine Proteins**		
Q91XA9	Acidic mammalian chitinase	Chia
Q6PZE0	Mucin-19	Muc19
Q8C6C9;D3YTP1	Protein LEG1 homolog	Leg1;2310057J18Rik
Q3UU35;Q3TTY9	Ovostatin homolog	Ovos;BC048546
A0A1R3UFA0;P15949	Kallikrein 1-related peptidase b9	Klk1b9
A0A1R3UGI5;P07628	Kallikrein 1-related peptidase b8	Klk1b8
Q61114	BPI fold-containing family B member 1	Bpifb1
B1AVU4	Predicted gene 14744	Gm14744
A0A1R3UDC2;P36369	Kallikrein 1-related peptidase b26	Klk1b26
Q91WL7;Q14BW7	Deoxyribonuclease	Dnase1
Q5SW46;Q91WA0	Lactoperoxidase	Lpo
L7N1X9	Demilune cell and parotid protein 1	Dcpp1
A0A0B6VSQ6;P15947	Kallikrein-1	Klk1
L7N259	Demilune cell and parotid protein 3	Dcpp3
Q9CPP7	Gastric triacylglycerol lipase	Lipf
E9PYC2	Demilune cell and parotid protein 2	Dcpp2
A0A1R3UGK0;P00757	Kallikrein 1-related peptidase-like b4	Klk1b4
Q3V469;Q9JHY3	WAP four-disulfide core domain protein 12	Wfdc12
P11591	Major urinary protein 5	Mup5
Q8VC95;Q9EQG0	Mucin cell adhesion protein	Prol1
P08071;Q4FJR3	Lactotransferrin	Ltf
Q3UTR7;P11859	Angiotensinogen	Agt
D3YYY1	Androgen binding protein BG7	Scgb2b7
S4R244;Q544L5	Prostatic spermine-binding protein	Sbp
A2BHR2	Lipocalin 11	Lcn11
Q8VD07;P01132	Pro-epidermal growth factor; Epidermal growth factor	Egf
Q8BND5	Sulfhydryl oxidase 1	Qsox1
Q3TWM9;E9PZ00	Prosaposin	Psap
O70570	Polymeric immunoglobulin receptor; Secretory component	Pigr
Q3UCD9;Q3U7P0	Cathepsin D	Ctsd
A0A0R4J043;P28825	Meprin A subunit alpha; Metalloendopeptidase	Mep1a
P05533	Lymphocyte antigen 6A-2/6E-1	Ly6a

**Sweat Nest and Urine Proteins**		
C1KG51;A0A0A6YVU7	Truncated profilaggrin/filaggrin flaky tail mutant form	Flg
Q3MI12;Q9D6T8	2310057N15Rik protein (Fragment)	2310057N15Rik
Q91VB8;Q9CY10	Hemoglobin subunit alpha	haemaglobin alpha 2;Hbat1
Q5FWB7;P05064	Fructose-bisphosphate aldolase	Aldoa
G5E8Z3	MCG120169	2310050C09Rik
P52480;A0A1L1SU37	Pyruvate kinase PKM	Pkm
P11589	Major urinary protein 2	Mup2
Q58EV3;E9QA79	Major urinary protein 1; Major urinary protein 7	Mup1;Mup7
P22599	Alpha-1-antitrypsin 1-2	Serpina1b
A0A0R4J0I1;P07759	Serine protease inhibitor A3K	Serpina3k
A2CEK7	Major urinary protein 12	Mup14
P08228	Superoxide dismutase [Cu-Zn]	Sod1
**Proteins in 2 sample types**		
Protein IDs	Protein names	Gene names
**Saliva and Sweat Proteins**		
B2RTM0;P62806	Histone H4	Hist2h4;Hist1h4a
P01027	Complement C3	C3
Q00898	Alpha-1-antitrypsin 1-5	Serpina1e
P23953	Carboxylesterase 1C	Ces1c
**Saliva and Nest Proteins**		
B7ZCG3;P07743	BPI fold-containing family A member 2	Bpifa2
A0A1R3UCH4;P04071	Kallikrein 1-related peptidase b16	Klk1b16
Q540N3;P15948	Kallikrein 1-related peptidase b22	Klk1b22
Q8JZX1;Q7M745	Androgen binding protein BG26	Scgb2b26
A0A1R3UHM9;P00756	Kallikrein 1-related peptidase b3	Klk1b3
Q8K1H9	Odorant-binding protein 2a	Obp2a
B9EKG3;Q9Z0L8	Gamma-glutamyl hydrolase	Ggh
F6URP1	Predicted gene 6619	Gm6619
A0A1R3UCH5;Q9JM71	Kallikrein 1-related peptidase b27	Klk1b27
A0A1R3UCH6;P00755	Kallikrein 1-related peptidase b1	Klk1b1
Q545H0;Q03401	Cysteine-rich secretory protein 1	Crisp1
A0A1R3UDS6;P15946	Kallikrein 1-related peptidase b11	Klk1b11
A2BHR0	Odorant-binding protein 2b	Obp2b
A0A0G2JEK0;Q6LDU8	Beta-nerve growth factor	Ngf
Q80XI7	Vomeromodulin	Vom
E9QPG8;A0A140LI59	Deleted in malignant brain tumors 1 protein	Dmbt1
D2XZ39;Q7M747	Secretoglobin family 2B member 24	Scgb2b24
Q66VB7	Lacrein	Gm1553
A0A075B6A3;A0A0A6YXW6	Ig alpha chain C region	Igha;Igh;Igh-VJ558
Q3UP42;P31725	Protein S100-A9	S100a9

A8R0U8;A8R0U7	Exocrine gland secreted peptide 15	Esp15
**Saliva and Urine Proteins**		
E9Q5I3;Q8K1G6	Mucin 5, subtype B, tracheobronchial	Muc5b
Q9JM84	Cystatin 10	Cst10
Q3UKN6;P81117	Nucleobindin-2;Nesfatin-1	Nucb2
Q549A5;Q06890	Clusterin beta chain; Clusterin alpha chain	Clu
Q9D6Y8;Q9CPP2	Uncharacterized protein	Sbpl
**Sweat and Nest Proteins**		
Q9D6L6	RIKEN cDNA 2310079G19 gene	2310079G19Rik
E9QPZ3;Q2VIS4	Filaggrin-2	Flg2
P18165	Loricrin	Lor
D3Z724	Predicted gene 5965	Gm5965
Q5SXZ7;A0A0A0MQG3	Integrator complex subunit 2	Ints2
Q3TB63;Q3UDS0	Heat shock cognate 71 kDa protein; Heat shock-related 70 kDa protein 2	Hspa8;Hspa2
Q7TPC1;Q3V0M9	Corneodesmosin	Cdsn
Q61171;D3Z4A4	Peroxiredoxin-2	Prdx2
Q61838	Alpha-2-macroglobulin	A2m
Q5M9K1;P07309	Transthyretin	Ttr
**Sweat and Urine Proteins**		
E9Q557;E9PZW0	Desmoplakin	Dsp
P09411;S4R2M7	Phosphoglycerate kinase 1	Pgk1
Q52L87;Q3TIZ0	Tubulin alpha-1C chain; Tubulin alpha-1A chain	Tuba1c;Tuba1a
P00920;A0A0A6YX78	Carbonic anhydrase 2	Ca2;Car2
Q5SVY2;Q3UAJ1	Peptidyl-prolyl cis-trans isomerase	Ppia
Q00897	Alpha-1-antitrypsin 1-4	Serpina1d
G3UVV4;Q6GQU1	Hexokinase	Hk1
**Urine and Nest Proteins**		
Q91X17	Uromodulin	Umod
B7ZNS9;Q3UP47	Complement factor D	Cfd
L7MUC7	Major urinary protein 7 (Fragment)	Mup7
Q9JM79;Q9DCS8	Napsin-A	Napsa
P35459	Lymphocyte antigen 6D	Ly6d
Q07456	Protein AMBP	Ambp
Q547B5;Q3TND2	Osteopontin	Spp1
Q6S9I0;Q6S9I2	Kininogen 2	Kng2
Q149Y8;Q08423	Trefoil factor 1	Tff1
P00688;Q8C5B4	Pancreatic alpha-amylase	Amy2;Amy2a1
B8JI96	Major urinary protein 14 (Fragment)	Mup14
Q91XL1	Leucine-rich HEV glycoprotein	Lrg1
O09114	Prostaglandin-H2 D-isomerase	Ptgds
Q102J0;Q8R242	Di-N-acetylchitobiase	Ctbs
Q3UDD6;Q544Y8	Granulins; Acrogranin	Grn

E9PVG8	RIKEN cDNA 9530053A07 gene	9530053A07Rik
Q3UBS3;Q61646	Haptoglobin alpha chain; Haptoglobin beta chain	Hp
P09036	Serine protease inhibitor Kazal-type 3	Spink3
**Unique Proteins**		
Protein IDs	Protein names	Gene names
**Saliva**		
Q61902;Q61900	Submaxillary gland androgen-regulated protein 3A	Smr3a
A0A1R3UCI2;P15945	Kallikrein 1-related peptidase b5	Klk1b5
Q3U3J1	2-oxoisovalerate dehydrogenase subunit	Bckdha
Q3TTT1;P18761	Carbonic anhydrase 6	Car6;Ca6
A0A2I3BRY2;P02815	16.5 kDa submandibular gland glycoprotein	Spt1
A0A1R3UCH3;Q61754	Kallikrein 1-related peptidase b24	Klk1b24
P97361	BPI fold-containing family A member 1	Bpifa1
Q3UKV9;Q06318	Uteroglobin	Scgb1a1
Q3UNG6;Q61759	Kallikrein 1-related peptidase b21	Klk1b21
Q91X93	Proline-rich protein BstNI subfamily 1	Prb1
B7ZWD8;A0MA77	Uncharacterized protein	Dcpp2
Q14AV3;Q8C1E1	BPI fold-containing family B member 2	Bpifb2
A0A077S2U6;P08905	Lysozyme C-2	Lyz2
Q4FK86;O88593	Peptidoglycan-recognition protein	Pglyrp1
Q24JQ8;Q62472	Vomeronasal secretory protein 2	Lcn4
Q545I1;O09049	Regenerating islet-derived protein 3-gamma	Reg3g
O88309;P36368	Epidermal growth factor-binding protein type B	Egfbp2
Q61297	Alpha-amylase	NA
E9PWS6	RIKEN cDNA A630073D07	A630073D07Rik
Q14AJ3;Q62471	Vomeronasal secretory protein 1	Lcn3
Q3UWH6;Q9D0C0	Cathepsin L1	Ctsl
Q9D7Y7;Q9CPN9	NA	2210010C04Rik
Q3TVS6;Q3TC17	Cathepsin B	Ctsb
A0A077S9N1;P17897	Lysozyme	Lyz1
Q80ZU7;Q3V181	BPI fold-containing family B member 3	Bpifb3
Q0VDQ3;A0A0R4J0B9	Pancreatic secretory granule membrane major glycoprotein GP2	Gp2
Q53X15;P27005	Protein S100;Protein S100-A8	S100a8
Q9CQV3	Serpin B11	Serpinb11
A0A1C7CYU3;Q8BRD3	Nucleobindin-1	Nucb1
Q8VEH9;Q3UQ05	cDNA sequence BC018465; BPI fold-containing family B, member 5	Bpifb5
Q07797;E9Q5X5	Galectin-3-binding protein	Lgals3bp
O35744	Chitinase-like protein 3	Chil3
O08692	Neutrophilic granule protein	Ngp
G5E8B5;Q7M742	Secretoglobin family 1C member 1	Scgb1c1

E9Q704;F8VQA4	Peptidyl-glycine alpha-amidating monooxygenase; Peptidylglycine alpha-hydroxylating monooxygenase	Pam
Q544T7;A0A1W2P788	alpha-1,2-Mannosidase	Man1a;Man1a1
Q5SXG7	Vitelline membrane outer layer protein 1 homolog	Vmo1
Q8BKY2	Uncharacterized protein	Col3a1
E9PWB6;E9QAQ8	Mucin 5, subtypes A and C, tracheobronchial/gastric	Muc5ac
P21956;Q3TDU5	Lactadherin	Mfge8
F8WHM5;Q53WR6	Golgi apparatus protein 1	Glg1
Q3UDR2;Q3URP6	Protein disulfide-isomerase	P4hb
Q8BG86;G3X9V8	NA	Serpinb3a;Scca2; Serpinb3c
Q3UBP6;Q3UBQ4	NA	Actb
Q3TYW1;O55226	Chondroadherin	Chad
**Sweat**		
A5JUZ1;A0A0A6YW67	Ubiquitin-60S ribosomal protein L40; Ubiquitin-40S ribosomal protein S27a	Ubc;Gm8797
Q9D746	RIKEN cDNA 2310034C09	2310034C09Rik
Q02257	Junction plakoglobin	Jup
Q9D6S9	NA	2310061N02Rik
P17751;H7BXC3	Triosephosphate isomerase	Tpi1
Q9QUK9;Q3V2E0	MCG15083; Uncharacterized protein	Try5;Try4
Q8CE60;B2RXW1	Histidine ammonia-lyase	Hal
B2RQH0;Q7TSF1	Desmoglein-1-beta;Desmoglein-1-alpha	Dsg1b;Dsg1a
Q6WEH7;Q9JM83	Calmodulin-4	Calm4
Q9D7K4;Q9CRB1	Galectin;Galectin-7	Lgals7
A0JLR7;Q61484	Ahnak protein (Fragment); Desmoyokin (Fragment)	Ahnak
Q9D6U7;A2RTA0	Creatine kinase M-type	Ckm
Q9CZI7;Q542G9	Annexin A2	Anxa2
Q8VEE3;A0JLV3	Histone H2B	Hist2h2bb
P97350	Plakophilin-1	Pkp1
A0A0R4J293;Q9JLF6	Protein-glutamine gamma-glutamyltransferase K	Tgm1
Q3U7Z6;Q9DBJ1	Phosphoglycerate mutase 1	Pgam1
Q62266	Cornifin-A	Sprr1a
Q62267	Cornifin-B	Sprr1b
Q8BLX1	Protein S100	Hrnr
Q3TU85;A1E2B8	Heat shock 70 kDa protein 1A; Heat shock 70 kDa protein 1B	Hspa1b;Hspa1a
A0A0A6YW46;A0A0A6YX57	Filaggrin	Flg
Q8C605;Q9WUA3	ATP-dependent 6-phosphofructokinase	Pfkp
P40142;A0A286YE28	Transketolase	Tkt
Q6P6I3;Q91YH6	ATPase, H+ transporting, lysosomal V1 subunit B1	Atp6v1b1

Q6PAC1;Q3U9Q8	Gelsolin	Gsn
P21614	Vitamin D-binding protein	Gc
P50516	V-type proton ATPase catalytic subunit A	Atp6v1a
Q4FK88;Q4FJV4	Annexin A1	Anxa1
Q71LX8;P11499	Heat shock protein HSP 90-beta	Hsp90ab1
Q3TE06;Q3TNK2	WD repeat-containing protein 1	Wdr1
P14152	Malate dehydrogenase, cytoplasmic	Mdh1
Q548W7;P31786	Acyl-CoA-binding protein	Dbi
Q4FJX4;P97315	Cysteine and glycine-rich protein 1	Csrp1
P28665	Murinoglobulin-1	Mug1
G3X9T8;G3X8Q5	Ceruloplasmin	Cp
Q5HZY7;Q9CR51	V-type proton ATPase subunit G 1	Atp6v1g1
A0A338P7B8;Q6YJU1	Fetuin-B	Fetub
Q3ULT2;Q3UDJ7	Alpha-actinin-4	Actn4
Q6ZWX2	Thymosin, beta 4, X chromosome	Tmsb4x
A0A0A6YXG4	Filaggrin	Flg
Q99PT1	Rho GDP-dissociation inhibitor 1	Arhgdia
D3YTY9;A0A0R4J038	Kininogen-1	Kng1
A0A075B5P4;A0A0A6YWR2	Ig gamma-1 chain C region	Ighg1;HC
P21550;Q4FK59	Beta-enolase; Enolase	Eno3
P12382;Q8CD98	ATP-dependent 6-phosphofructokinase, liver type	Pfkl
Q5EBQ2;Q3TGC5	Phosphatidylethanolamine-binding protein 1	Pebp1
Q8CBU4;Q4KML7	Ezrin	Ezr
Q9CWS5	Uncharacterized protein	Uncharacterized protein
Q61509;Q99LT6	Elongation factor 2	Eef2
Q3U6E4;Q0VGU2	Prothymosin alpha; Thymosin alpha	Ptma;Gm12504
Q01853;Q8BNF8	Transitional endoplasmic reticulum ATPase	Vcp
Q60829	Protein phosphatase 1 regulatory subunit 1B	Ppp1r1b
Q545F0;P34884	Macrophage migration inhibitory factor	Mif
Q3TG37;P70441	Na(+)/H(+) exchange regulatory cofactor NHE-RF	Slc9a3r1
Q8BPH1;Q5SS40	14-3-3 protein epsilon	Ywhae
Q66JR8;Q9D0J8	Parathymosin	Ptms
P35700;B1AXW5	Peroxiredoxin-1	Prdx1
Q9QXD6;Q9QXC5	Fructose-1,6-bisphosphatase 1	Fbp1
Q3TZ44;Q3TSZ4	Aldose 1-epimerase	Galm
D3Z7F0;P16125	L-lactate dehydrogenase; L-lactate dehydrogenase B chain	Ldhb
Q544Y7;F8WGL3	Cofilin-1;Cofilin-2	Cfl1;Cfl2
Q91YT9;Q91V28	6-phosphogluconate dehydrogenase, decarboxylating	Pgd

**Nest**		
E9Q328;Q9D3N7	RIKEN cDNA 5430401F13 gene	5430401F13Rik
J3QK77;Q9JI02	Secretoglobin family 2B member 20	Scgb2b20
J3QME6;F6WYC8	Lipase	Gm5097
Q80XE3	BC051076 protein (Fragment)	BC051076
Q9D7P9	Serpin B12	Serpinb12
A8R0U0	Exocrine gland secreted peptide 6	Esp6
J3QJY4	Androgen binding protein A3	Scgb1b3
D3Z617;D3Z4E7	Seminal vesicle antigen-like 2	Sval2
S4R2L0;J3QM75	Androgen binding protein BG12; Androgen binding protein BG19	Scgb2b12;Scgb2b19
Q3UWK8	MCG20280	Serpinb6d
Q32ME9;Q2VPA9	Desmocollin-1	Dsc1
E9Q9C6;E9Q0B5	Fc fragment of IgG-binding protein	Fcgbp
Q9D3N5	RIKEN cDNA 5430402E10 gene	5430402E10Rik
Q0VGU8	BPI fold-containing family A, member 6	Bpifa6
Q7TT08;Q3UXH6	Lipase	Lipo1
Q9ES55;Q3TYQ9	Aldehyde oxidase 4	Aox4
Q3UW77;B9EKG4	MCG59630; Predicted gene, OTTMUSG00000008911	Gm12888
B1AVM1	Predicted gene 12887	Gm12887
Q0VDV3;W0UVC5	Ribonuclease 2B	Rnase2b
Q4KL81;Q3TSB7	Actin, cytoplasmic 2	Actg1
Q9D0H8	Uncharacterized protein	Uncharacterized protein
Q9QZ83	Gamma actin-like protein	Actg1
O88312	Anterior gradient protein 2 homolog	Agr2
P01592	Immunoglobulin J chain	Igj
O09133	Submaxillary gland androgen-regulated protein 2, isoform alpha	Smr2
S4R1X8;S4R2V3	Secretoglobin, family 2B, member 17; member 15	Scgb2b17;Scgb2b15
Q9ET22	Dipeptidyl peptidase 2	Dpp7
A0A0R4J077;Q8R1M8	Mucosal pentraxin	Mptx1
A0A089N3F1;D2XZ38	Androgen binding protein BG3	Abpbg3;Scgb2b3
O09131	Glutathione S-transferase omega-1	Gsto1
**Urine**		
Q80YV5;Q9QX97	Trefoil factor 2	Tff2
P11087	Collagen alpha-1(I) chain	Col1a1
A0A0N4SV66;A0AUV1	Histone H2A	Hist1h2ah
A9R9V7	Major Urinary Protein 21	Mup21
Q4KML8;A0A0R4IZW5	Cadherin-1	Cdh1
B7ZNZ9;Q02596	Glycosylation-dependent cell adhesion molecule 1	Glycam1
F6VHS4;Q5M9M1	Hepcidin-2	Hamp2
Q505K6;Q8BWN9	AI182371	AI182371

A0A087WRP7;A0A087 WNZ5	Lymphocyte antigen 6C1;Lymphocyte antigen 6C2	Ly6c1;Ly6c2
Q8BHC0	Lymphatic vessel endothelial hyaluronic acid receptor 1	Lyve1
Q8JZM3;Q78ZN4	Resistin-like alpha	Retnla;Xcp2
A0A0N4SWB4;Q3UQF0	Kidney androgen-regulated protein	Kap
Q8R1I3;E9Q6G4	ATP-binding cassette sub-family A member 7	Abca7
Q5XFY8;A0A0M3KL49	Ig kappa chain C region	Igkc
Q5SSJ1	Activated macrophage/microglia WAP domain protein	Wfdc17
A2CEK6;L7N222	Major urinary protein 11; Major urinary protein 13	Mup13
Q62395	Trefoil factor 3	Tff3
Q91X23;Q60590	Alpha-1-acid glycoprotein;Alpha-1-acid glycoprotein 1	Orm1
A2ARV4;A2ARV5	Low-density lipoprotein receptor-related protein 2	Lrp2
Q920X5;Q91VE7	Cathelin-related antimicrobial peptide	Cramp
P68372;Q9CVR0	Tubulin beta-4B chain; Tubulin beta-4A chain	Tubb4b;Tubb4a
P13634	Carbonic anhydrase 1	Ca1
Q53ZF0;P97426	Eosinophil cationic protein 1	Ear1;R8;R9;Ear10
Q0VBA8;P06869	Urokinase-type plasminogen activator	Plau
A0A140T8N2;P01642	Ig kappa chain V-V region L7	Gm10881

Table S1. Comprehensive list of all proteins detected across samples. Detected proteins had at least 2 MS/MS counts in two replicates of a single sample type. List is limited to the first two protein IDs where applicable and organized in descending order by how many sample types each protein was detected in.

Supplementary Table 2

Protein names	PC1 loading	PC1 contribution (%)	PC2 loading	PC2 contribution (%)
Alpha-amylase 1	**0.798322**	**1.15684669**	0.142616	0.059547001
Carbonic anhydrase 6	**0.807033**	**1.182231837**	-0.02831	0.002347222
BPI fold-containing family B, member 9B	**0.79865**	**1.157798817**	-0.00331	3.21E-05
ABPBG27; Salivary androgen-binding protein beta subunit	**0.768089**	**1.07088558**	-0.1989	0.115825627
Prolactin-inducible protein homolog	**0.866815**	**1.363870657**	0.27678	0.224282802
Major urinary protein 4	**0.678011**	**0.834436943**	**0.547831**	**0.878657058**
Odorant-binding protein 1a	0.15211	0.041998573	**-0.63503**	**1.180646659**
Serum albumin	-0.35751	0.232010156	0.242068	0.171553507
Predicted gene 14743	0.324471	0.191105056	**-0.51526**	**0.777271467**
Transcobalamin-2	0.62512	0.709328451	-0.41643	0.507698768
Androgen binding protein A2	-0.05893	0.006303273	**-0.54272**	**0.862351646**
Major urinary protein 1	-0.0091	1.50E-04	**0.859771**	**2.164170871**
Androgen binding protein A7; A20	**0.644876**	**0.754871328**	0.228754	0.153201892
Serotransferrin	-0.36535	0.24228555	**-0.57261**	**0.959925112**
Major urinary protein 10; Major urinary protein 1	-0.29127	0.153992106	0.446103	0.58263389
Major urinary protein 20	0.174673	0.055382503	**0.937846**	**2.575069199**
Hornerin	-0.3263	0.193266853	-0.4495	0.591544847
Beta-globin	-0.5646	0.578631526	-0.12396	0.044987337
Actin, cytoplasmic 1	-0.55587	0.56087682	0.188971	0.104547557
Glyceraldehyde-3-phosphate dehydrogenase	-0.48893	0.433925726	-0.06484	0.012308331
Elongation factor 1-alpha	-0.49026	0.436294231	-0.07763	0.017645381
Major urinary protein 3	0.33133	0.199270355	**0.712766**	**1.487372728**
Androgen binding protein A27	0.34612	0.217456495	**-0.75965**	**1.689484077**
Predicted gene 5938	-0.08825	0.014135456	**-0.68919**	**1.390600221**
Secretoglobin family 2B member 2	**0.65872**	**0.787629644**	-0.48153	0.678859812
Alpha-enolase;Enolase	-0.60585	0.666267069	**-0.53726**	**0.845071425**
Protein-glutamine gamma-glutamyltransferase E	-0.29773	0.160908094	**-0.60456**	**1.070039369**
Alpha-2-HS-glycoprotein	-0.03128	0.00177635	**-0.661**	**1.279156172**
Hemopexin	0.015665	4.45E-04	**-0.6079**	**1.081899659**
L-lactate dehydrogenase	**-0.69667**	**0.880986196**	-0.37775	0.41776742
Glucose-6-phosphate isomerase	-0.59852	0.650237244	-0.32389	0.307125481

Acidic mammalian chitinase	**0.832652**	**1.258481875**	0.143859	0.060589852
Mucin-19	**0.901206**	**1.474238245**	-0.1174	0.040349124
Protein LEG1 homolog	**0.922667**	**1.545288835**	0.369059	0.39876598
Ovostatin homolog	**0.968056**	**1.701064721**	0.216393	0.137092404
Kallikrein 1-related peptidase b9	**0.850287**	**1.312353612**	-0.26031	0.19838195
Kallikrein 1-related peptidase b8	**0.842484**	**1.288377231**	-0.24739	0.17918074
BPI fold-containing family B member 1	**0.769847**	**1.075792894**	-0.12773	0.047768334
Predicted gene 14744	**0.849526**	**1.310005399**	-0.05074	0.00753626
Kallikrein 1-related peptidase b26	**0.822848**	**1.229021007**	-0.20659	0.124949633
Deoxyribonuclease	**0.90787**	**1.496122544**	0.40255	0.474421737
Lactoperoxidase	**0.927525**	**1.561603888**	0.36651	0.393275481
Demilune cell and parotid protein 1	**0.876866**	**1.395683456**	-0.04863	0.006923083
Kallikrein-1	**0.857968**	**1.336170512**	**0.506277**	**0.750415338**
Demilune cell and parotid protein 3	**0.908707**	**1.49888248**	0.409404	0.490714537
Gastric triacylglycerol lipase	**0.775113**	**1.090560163**	-0.11078	0.035930841
Demilune cell and parotid protein 2	**0.87878**	**1.401782637**	0.175806	0.090488276
Kallikrein 1-related peptidase-like b4	**0.833005**	**1.259550085**	-0.22457	0.147647091
WAP four-disulfide core domain protein 12	**0.869038**	**1.370875523**	-0.26994	0.213328528
Major urinary protein 5	**0.911506**	**1.508132167**	0.388251	0.441317856
Mucin cell adhesion protein	**0.853592**	**1.322575741**	-0.23187	0.157406887
Lactotransferrin	**0.892698**	**1.446535908**	0.134573	0.053020202
Angiotensinogen	**0.891231**	**1.441786403**	-0.09209	0.024826185
Androgen binding protein BG7	**0.85892**	**1.33913761**	**0.502101**	**0.738086804**
Prostatic spermine-binding protein	**0.865221**	**1.358858326**	0.186544	0.101880275
Lipocalin 11	**0.81701**	**1.211642375**	-0.19221	0.108163199
Pro-epidermal growth factor; Epidermal growth factor	**0.807961**	**1.184951062**	**0.581342**	**0.989437794**
Sulfhydryl oxidase 1	**0.83595**	**1.268470601**	-0.23429	0.160710506
Prosaposin	0.62519	0.709487314	**0.637878**	**1.191246016**
Polymeric immunoglobulin receptor; Secretory component	**0.843088**	**1.29022527**	-0.21252	0.132223189
Cathepsin D	**0.848946**	**1.308218179**	-0.19799	0.114761645
Meprin A subunit alpha; Metalloendopeptidase	0.25558	0.118569245	**0.838775**	**2.059762369**
Lymphocyte antigen 6A-2/6E-1	0.201072	0.073387737	**0.784296**	**1.800884982**
Truncated profilaggrin/filaggrin flaky tail mutant form	**-0.63868**	**0.740429271**	-0.17796	0.092715738
2310057N15Rik protein (Fragment)	**-0.67574**	**0.828854885**	0.026208	0.002010906
Hemoglobin subunit alpha	**-0.64906**	**0.764694443**	-0.19514	0.111481134
Fructose-bisphosphate aldolase	**-0.74145**	**0.997885559**	-0.2107	0.129969129
MCG120169	**-0.6269**	**0.713372973**	-0.16536	0.080055323

Pyruvate kinase PKM	**-0.825**	**1.235444881**	0.148551	0.064606836
Major urinary protein 2	-0.03919	0.002788514	**0.616163**	**1.111518946**
Major urinary protein 1; Major urinary protein 7	**-0.64358**	**0.751850694**	**0.629222**	**1.159135101**
Alpha-1-antitrypsin 1-2	-0.51566	0.482663155	**0.621607**	**1.131247661**
Serine protease inhibitor A3K	-0.518	0.487065878	**0.583216**	**0.995830346**
Major urinary protein 12	-0.07423	0.010000922	**0.839573**	**2.063683923**
Superoxide dismutase [Cu-Zn]	-0.47599	0.411265657	0.398747	0.465500096
Histone H4	0.173373	0.054561102	-0.44052	0.568130791
Complement C3	-0.33374	0.202180757	**-0.60396**	**1.067931254**
Alpha-1-antitrypsin 1-5	-0.13319	0.032198333	-0.33425	0.327086412
Carboxylesterase 1C	-0.13501	0.033085447	**-0.52901**	**0.819328525**
BPI fold-containing family A member 2	**0.86847**	**1.369082488**	-0.46544	0.634227176
Kallikrein 1-related peptidase b16	**0.85593**	**1.329829823**	-0.41881	0.513534887
Kallikrein 1-related peptidase b22	**0.855381**	**1.32812677**	-0.41833	0.51235487
Androgen binding protein BG26	**0.863623**	**1.353842297**	-0.43996	0.566711385
Kallikrein 1-related peptidase b3	**0.856219**	**1.330729125**	-0.42029	0.517163282
Odorant-binding protein 2a	**0.856791**	**1.332509366**	-0.42036	0.517328699
Gamma-glutamyl hydrolase	**0.793306**	**1.142357326**	-0.46584	0.635340674
Predicted gene 6619	**0.842855**	**1.289512571**	-0.39305	0.452286286
Kallikrein 1-related peptidase b27	**0.852914**	**1.320474863**	-0.4116	0.496005121
Kallikrein 1-related peptidase b1	0.531028	0.511863647	-0.24953	0.182292712
Cysteine-rich secretory protein 1	**0.7908**	**1.135150604**	**-0.49248**	**0.710074489**
Kallikrein 1-related peptidase b11	**0.856511**	**1.331637799**	-0.42087	0.518597672
Odorant-binding protein 2b	**0.852506**	**1.319212303**	-0.4114	0.495520507
Beta-nerve growth factor	0.3787	0.260321756	-0.14826	0.064352957
Vomeromodulin	**0.854462**	**1.325273778**	-0.4166	0.508113327
Deleted in malignant brain tumors 1 protein	**0.856042**	**1.330178804**	-0.42074	0.5182785
Secretoglobin family 2B member 24	0.575655	0.601513394	-0.15288	0.068424053
Lacrein	0.417484	0.316373716	-0.05883	0.010131111
Ig alpha chain C region	**0.692651**	**0.870860647**	-0.27956	0.228813115
Protein S100-A9	0.495082	0.444912088	-0.28099	0.231150303
Exocrine gland secreted peptide 15	0.411483	0.307342782	-0.04665	0.006372509
Mucin 5, subtype B, tracheobronchial	**0.673465**	**0.823285599**	0.017405	8.87E-04
Cystatin 10	**0.671375**	**0.818182183**	-0.29831	0.260539104
Nucleobindin-2;Nesfatin-1	**0.707252**	**0.907963871**	-0.30959	0.280615579
Clusterin	0.235572	0.10073187	**0.643805**	**1.213484521**
Uncharacterized protein	0.527721	0.505508759	-0.01616	7.65E-04
RIKEN cDNA 2310079G19 gene	-0.42739	0.331558261	-0.2418	0.1711752
Filaggrin-2	-0.60729	0.669445166	-0.3453	0.349067855
Loricrin	**-0.62553**	**0.710250252**	-0.35459	0.368114613

Predicted gene 5965	-0.0209	7.93E-04	-0.03731	0.004074735
Integrator complex subunit 2	**-0.73942**	**0.992434987**	-0.41808	0.511737432
Heat shock cognate 71 kDa protein;Heat shock-related 70 kDa protein 2	-0.39639	0.285212371	-0.24603	0.177210661
Corneodesmosin	-0.6108	0.677196769	-0.34655	0.351613214
Peroxiredoxin-2	-0.58429	0.619697259	-0.33328	0.325198177
Alpha-2-macroglobulin	-0.45466	0.375233548	-0.25858	0.195750513
Transthyretin	-0.41426	0.311505297	-0.23364	0.15981711
Desmoplakin	**-0.86613**	**1.361729454**	-0.22714	0.151043503
Phosphoglycerate kinase 1	**-0.87123**	**1.377791227**	-0.0508	0.007556025
Tubulin alpha-1C chain;Tubulin alpha-1A chain	**-0.84218**	**1.287435024**	0.423466	0.52500384
Carbonic anhydrase 2	-0.51531	0.482006062	-0.02119	0.001313978
Peptidyl-prolyl cis-trans isomerase	**-0.88572**	**1.424017049**	-0.08131	0.019354847
Alpha-1-antitrypsin 1-4	-0.27331	0.135590788	**0.776624**	**1.765821677**
Hexokinase	**-0.69366**	**0.873393534**	-0.16887	0.083493953
Uromodulin	0.118092	0.025313874	**0.943482**	**2.606109041**
Complement factor D	0.115793	0.024338047	**0.947204**	**2.626712567**
Major urinary protein 7 (Fragment)	0.122109	0.027065532	**0.933683**	**2.552256556**
Napsin-A	0.1229	0.027417263	**0.936562**	**2.568025124**
Lymphocyte antigen 6D	0.118445	0.025465668	**0.942604**	**2.601263541**
Protein AMBP	0.125464	0.028573311	**0.932924**	**2.548109747**
Osteopontin	0.117186	0.024926938	**0.945196**	**2.615587068**
Kininogen 2	0.123403	0.027642022	**0.936499**	**2.567675991**
Trefoil factor 1	0.12521	0.028457574	**0.933246**	**2.549867896**
Pancreatic alpha-amylase	0.130099	0.030723112	**0.926892**	**2.515266181**
Major urinary protein 14 (Fragment)	0.153852	0.042966317	**0.729679**	**1.558798648**
Leucine-rich HEV glycoprotein	0.126879	0.029221515	**0.930596**	**2.53541066**
Prostaglandin-H2 D-isomerase	0.123802	0.027820984	**0.935712**	**2.563364042**
Di-N-acetylchitobiase	-0.04289	0.003339061	**0.625016**	**1.143690016**
Granulins;Acrogranin	-0.04292	0.003343619	**0.62503**	**1.143741833**
RIKEN cDNA 9530053A07 gene	0.181213	0.059606895	**0.54118**	**0.857452315**
Haptoglobin	0.144224	0.037756911	**0.898921**	**2.365750669**
Serine protease inhibitor Kazal-type 3	0.133131	0.032172072	**0.919417**	**2.474862261**

Table S2. List of loading values and contributions for proteins on the first two principal components (PC). Log_2 LFQ intensities for the 140 proteins common to at least two sample types were used in the principal component analysis. Bolded values signify loadings with higher than expected contribution to each PC as determined by the square of the loading divided by the sum of the square of all loadings on each PC.

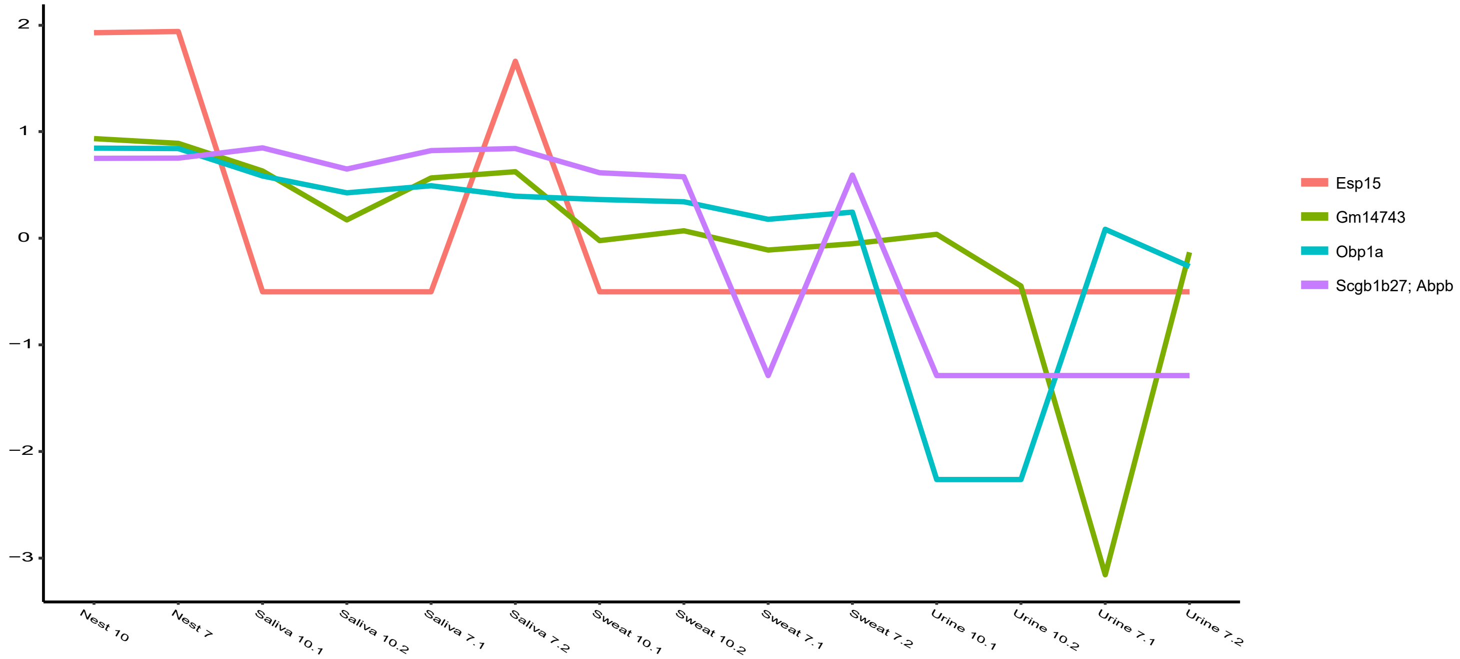

2
1
0
−1
−2
−3
Nest 10
Nest 7
Saliva 10.1
Saliva 10.2
Saliva 7.1
Saliva 7.2
Sweat 10.1
Sweat 10.2
Sweat 7.1
Sweat 7.2
Urine 10.1
Urine 10.2
Urine 7.1
Urine 7.2
Esp15
Gm14743
Obp1a
Scgb1b27; Abpb

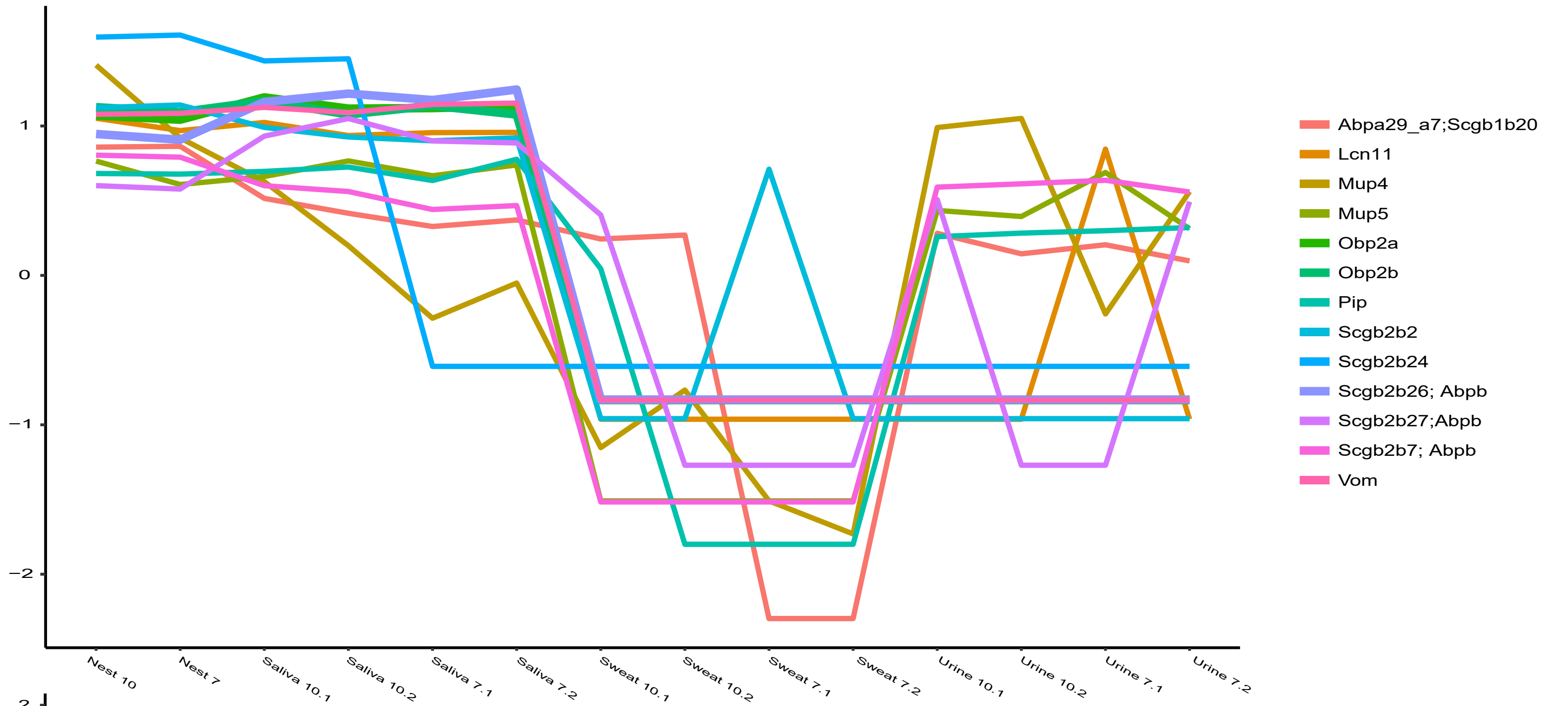

1
0
−1
−2
Nest 10
Nest 7
Saliva 10.1
Saliva 10.2
Saliva 7.1
Saliva 7.2
Sweat 10.1
Sweat 10.2
Sweat 7.1
Sweat 7.2
Urine 10.1
Urine 10.2
Urine 7.1
Urine 7.2
Abpa29_a7;Scgb1b20
Lcn11
Mup4
Mup5
Obp2a
Obp2b
Pip
Scgb2b2
Scgb2b24
Scgb2b26; Abpb
Scgb2b27;Abpb
Scgb2b7; Abpb
Vom

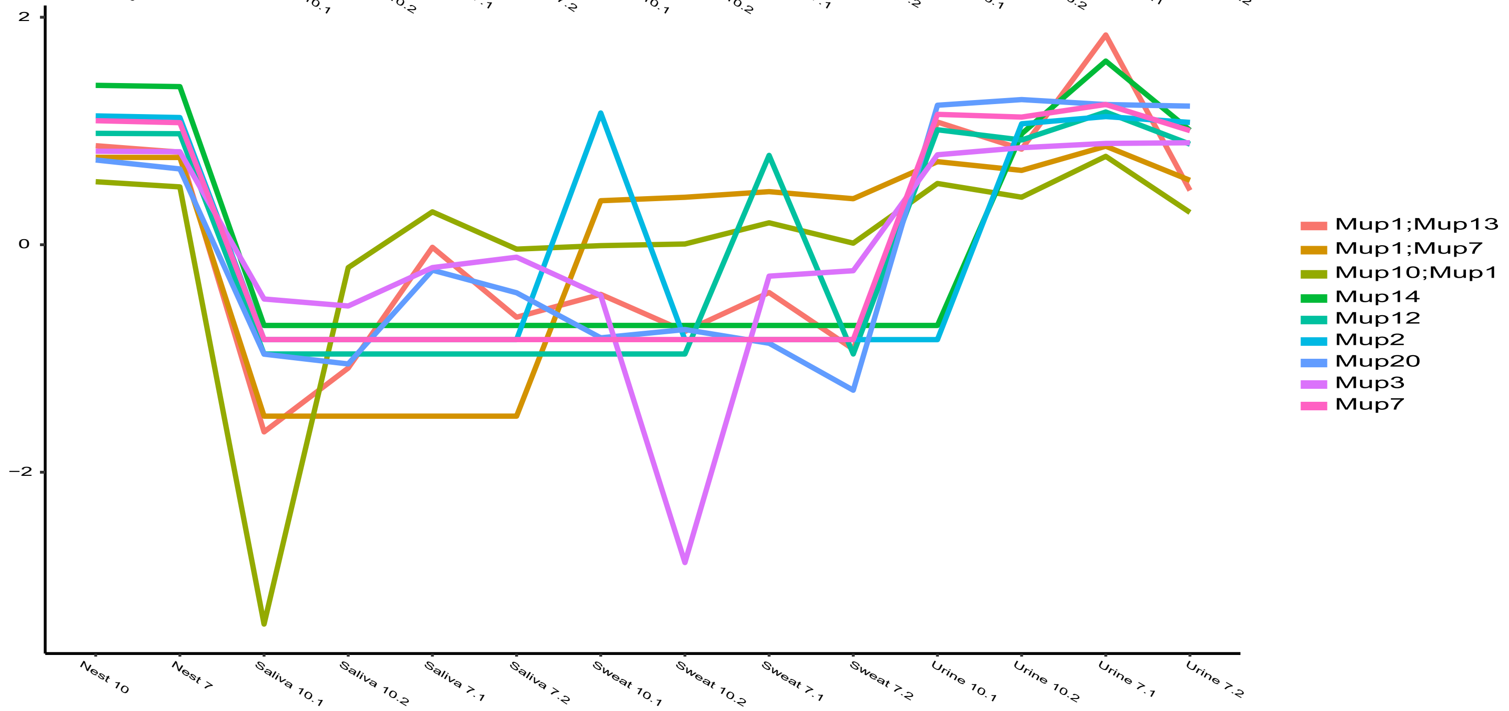

2
0
−2
Nest 10
Nest 7
Saliva 10.1
Saliva 10.2
Saliva 7.1
Saliva 7.2
Sweat 10.1
Sweat 10.2
Sweat 7.1
Sweat 7.2
Urine 10.1
Urine 10.2
Urine 7.1
Urine 7.2
Mup1;Mup13
Mup1;Mup7
Mup10;Mup1
Mup14
Mup12
Mup2
Mup20
Mup3
Mup7

Figure S1. Log_2 LFQ intensity expression patterns for 26 odorant proteins found across three hierarchical clusters as described in Figure 3.
